# Prognostic significance of three‐tiered pathological classification for microvascular invasion in patients with combined hepatocellular‐cholangiocarcinoma following hepatic resection

**DOI:** 10.1002/cam4.5328

**Published:** 2022-11-10

**Authors:** Yijun Wu, Hongzhi Liu, Yifan Chen, Jianxing Zeng, Qizhen Huang, Jinyu Zhang, Yongyi Zeng, Jingfeng Liu

**Affiliations:** ^1^ Department of Hepatobiliary Surgery Mengchao Hepatobiliary Hospital of Fujian Medical University Fuzhou People's Republic of China; ^2^ Shengli Clinical Medical College of Fujian Medical University; ^3^ Fujian Medical University Cancer Hospital & Fujian Cancer Hospital Fuzhou Fujian People's Republic of China; ^4^ The Big Data Institute of Southeast Hepatobiliary Health Information Mengchao Hepatobiliary Hospital of Fujian Medical University Fuzhou People's Republic of China

**Keywords:** combined hepatocellular‐cholangiocarcinoma (cHCC), grading, hepatic resection, microvascular invasion, pathology, prognosis

## Abstract

**Background and Objectives:**

Previous studies have reported that the microvascular invasion three‐tiered grading (MiVI‐TTG) scheme is a better prognostic predictor than the two‐tiered microvascular invasion (MiVI) grading scheme in hepatocellular carcinoma. This study aims to explore the prognostic significance of MiVI‐TTG in patients undergoing liver resection for combined hepatocellular‐cholangiocarcinoma (cHCC) and to explore the risk factors for MiVI in cHCC.

**Methods:**

This research included 208 patients graded as M0, M1, or M2 using the MiVI‐TTG scheme. Predictive performance was assessed by Cox regression analysis, Kaplan–Meier curve with Log rank test, Harrell's c‐index, and time‐dependent areas under the receiver operating characteristic curve (tdAUC). The clinical utility of the two schemes was evaluated by decision cure analysis (DCA). The risk factors for MiVI were evaluated using logistic regression analysis.

**Results:**

Among 208 cHCC patients, the proportions of M0, M1 and M2 were 38.9%, 36.5%, and 24.5%, respectively. Patients with severe MiVI status had worse recurrence‐free survival and overall survival (OS) based on Kaplan–Meier analysis. M1, M2, and MiVI‐positive were independent risk factors for early recurrence, while M2 and MiVI‐positive were associated with overall survival (OS). MiVI‐TTG had a larger c‐index, tdAUC, and net benefit rate than the two‐tiered MiVI grading scheme for predicting recurrence free survival and OS. AFP≥400 ng/ml was the independent risk factor for MiVI, and satellite nodules were independent risk factors for M2.

**Conclusions:**

MiVI‐TTG has a greater prognostic value than the two‐tiered MiVI grading scheme in patients undergoing hepatic resection for cHCC.

## Graphical abstract

The three‐tiered microvascular invasion (MiVI) grading scheme proposed by Liver Cancer Pathology Group of China showed greater prognostic value than the two‐tiered MiVI grading scheme in combined hepatocellular‐cholangiocarcinoma.

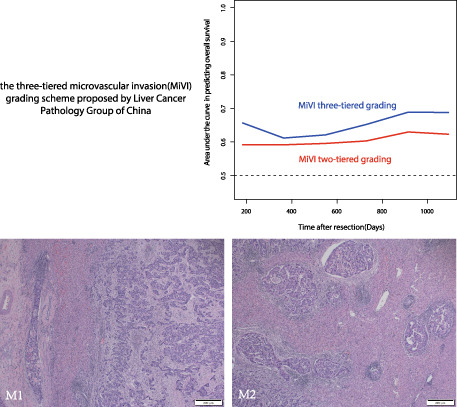

## INTRODUCTION

Combined hepatocellular cholangiocarcinoma (cHCC) is a rare subtype, accounting for only 0.4–14.2% of primary liver carcinomas (PLC).^1^, ^2^, ^3^, ^4^ Surgical resection is the first‐line treatment for cHCC.^1^, ^2^, ^3^, ^4^ Previous studies have reported that cHCC patients still suffered a dismal prognosis after hepatic resection, with a high recurrence rate and poor survival rate.^1^, ^2^, ^3^, ^4^ Owing to its rarity and wide variety of pathological types, research on cHCC has been challenging as the clinicopathologic characteristics and prognosis of this disease being poorly understood. In the 8th edition of the American Joint Committee on Cancer (AJCC) staging manual, the staging system of cHCC was designed in accordance with that of ICC, instead of being designed individually according to its own pathological and prognostic features.^5^ However, previous studies have shown significant differences between the clinicopathological characteristics and prognosis of cHCC and ICC,^1^, ^3^ which indicates the need for further research to explore the clinicopathological characteristics and prognosis of cHCC patients.

Microvascular invasion (MiVI) reflects aggressive biological behaviors of the tumor and has great predictive value for prognosis in PLC.^4^, ^6^, ^7^, ^8^, ^9^, ^10^ The incidence rate of MiVI, which varies from 30% to 68.7%, correlates with poor prognosis in patients with cHCC.^4^, ^6^, ^11^, ^12^, ^13^, ^14^, ^15^ Previous studies have shown that constructing individualized surveillance, such as wide‐margin hepatic resection, TACE, and antiviral treatment for patients with MiVI‐positivity or at high risk of recurrence after surgery for HCC significantly improves the OS and RFS.^9^, ^10^, ^16^ Therefore, the precise identification of MiVI is beneficial in constructing individualized surveillance strategies, thus improving the prognosis. The definition and research of MiVI were originally based on HCC; therefore, the main research on MiVI is highly concentrated on HCC.^9^, ^10^, ^16^ As research continues, the prognostic significance of MiVI for patients with cHCC after surgery has been clarified.^4^, ^6^, ^12^, ^14^ However, research on MiVI in cHCC patients is still limited,^4^, ^6^, ^11^, ^12^, ^13^, ^14^, ^15^ and wider exploration of MiVI is valuable for the management of cHCC.^4^, ^6^, ^14^

Which classification and definition of MiVI can reflect the aggressive biological behaviors of PLC and be applicable in clinical practice? Since the concept of MiVI was introduced, different types of classifications and definitions have been proposed.^9^, ^17^, ^18^, ^19^, ^20^, ^21^, ^22^, ^23^ With the increasing understanding of MiVI, MiVI is currently defined as the presence of tumor cell nests in the portal vein, hepatic vein, or tumor capsular vessel lined by endothelium, and were visible only under microscopy. A classification of two‐tiered MiVI schemes (i.e., present or absent) has been widely accepted and applied by most researchers.^9^, ^17^, ^22^, ^24^ Based on this definition, the Liver Cancer Pathology Group of China (LCPGC) has proposed a three‐tiered MiVI grading scheme (MiVI‐TTG) for hepatocellular carcinoma (HCC), ICC, and cHCC.^17^ In a large multicenter study, Sheng et al. found that MiVI‐TTG had a greater prognostic value than the two‐tiered MiVI scheme in patients with HCC after surgery.^22^ To the best of our knowledge, most studies on cHCC have elucidated the prognostic significance of MiVI following the definition described above with a two‐tiered scheme.^4^, ^6^, ^11^, ^12^, ^13^, ^14^, ^15^ Whether MiVI‐TTG has a greater prognostic value than the two‐tiered MiVI scheme in patients with cHCC after surgery has not been verified.

Therefore, this study aimed to evaluate the prognostic value of MiVI‐TTG compared with the two‐tiered MiVI scheme in patients who underwent hepatic resection for cHCC. Additionally, we investigated the risk factors for MiVI in cHCC.

## PATIENTS AND METHODS

### Patients

**FIGURE 1:**
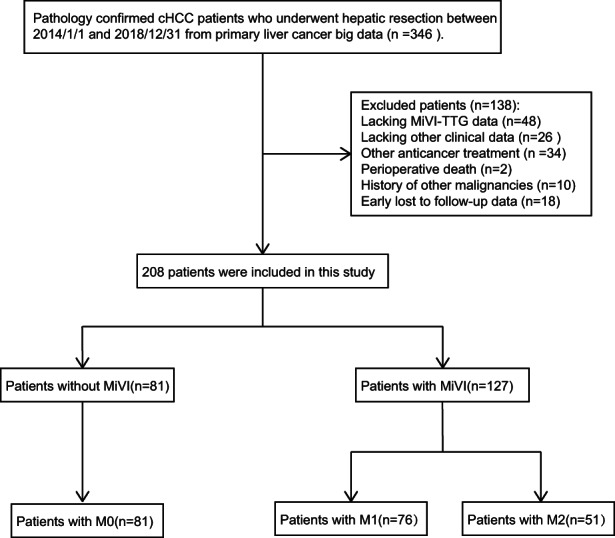
Flow chart of this study

In this retrospective study, 208 patients with cHCC who underwent hepatic liver resection between January 2014 and December 2018 were identified through primary liver cancer big data. The inclusion criteria were as follows: (a) pathologically confirmed cHCC (Allen type C) by hepatic resection, (b) Child‐Pugh A or B, and (c) macroscopic removal of all tumor nodules with a clear margin. Meanwhile, patients who met the following criteria were excluded: (a) prior anti‐tumor treatment, (b) loss of partial clinical data, (c) other malignancies or extrahepatic metastasis, and (d) failure to follow up over 12 months after resection. A flow chart of the selection of patients is shown (Figure 1).

### Hepatic resection

The type of hepatic resection was determined by the tumor size, tumor location, liver function, and preoperative diagnosis. Hepatic resection without lymph node dissection was performed preoperatively in the patients diagnosed with HCC. Hepatic resection with lymph node dissection was performed preoperatively in the patients diagnosed with ICC or cHCC. Extended hepatic resection was sometimes performed to guarantee tumor‐free resection margins. Lymph node dissection was performed when abnormally swollen lymph nodes were found behind the pancreatic head or around the hepatoduodenal ligament and common hepatic artery during surgery. Resection of three or more Couinaud segments was regarded as a major hepatic resection.

### Sampling protocol

**FIGURE 2:**
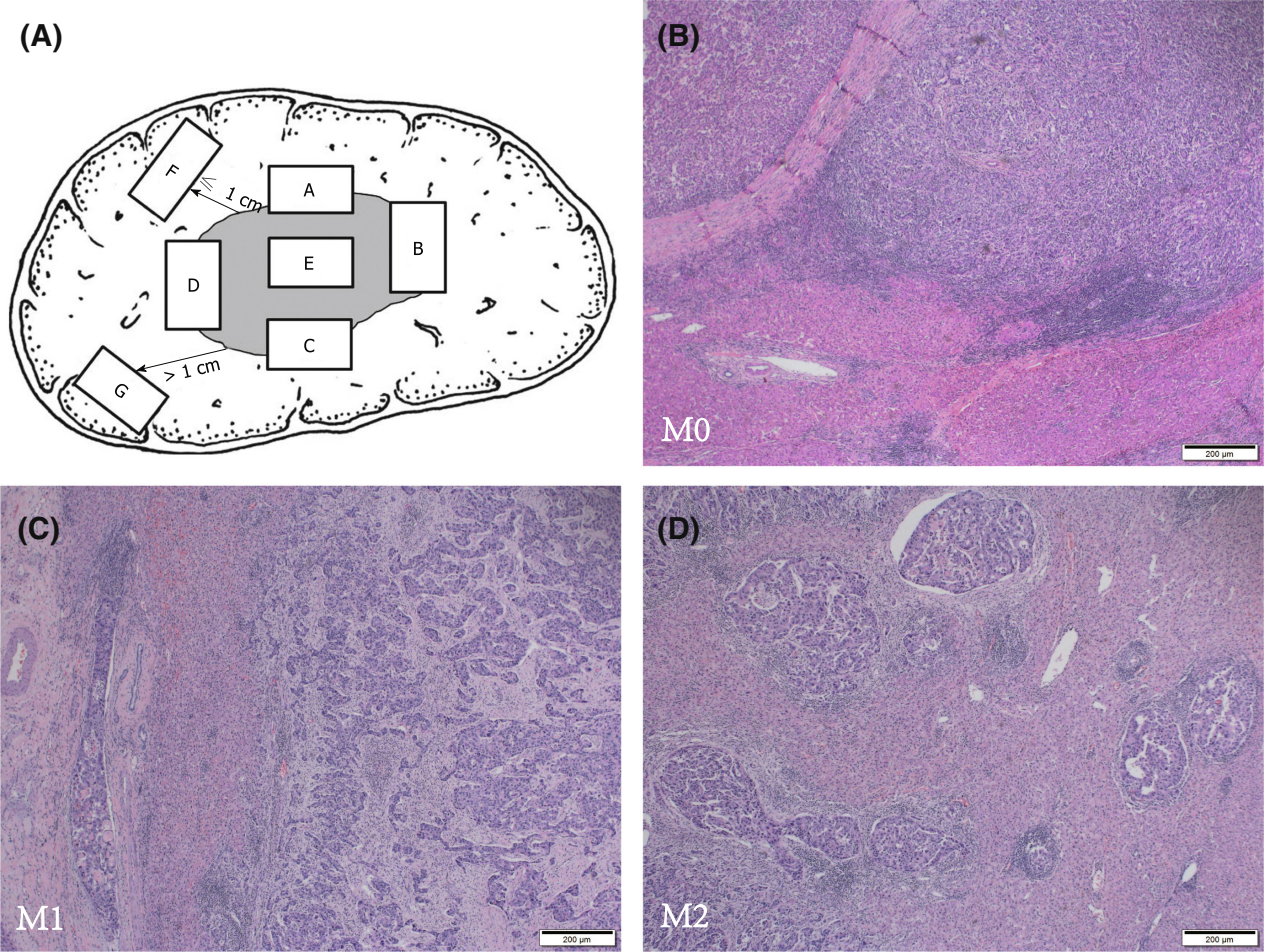
(A) 7‐point sample collection protocol; (B) M0 (no MiVI); (C) M1 (1–5 MiVIs existing ≤ 1 cm away from the tumor capsule); (D) M2 (>5 MiVIs, or any MiVI existing >1 cm away from the tumor capsule).

The specimens were collected according to 7‐point sample collection protocol described in “Practice guidelines for the pathological diagnosis of primary liver cancer: 2015 update” proposed by LCPGC (Figure 2A). At least one tissue was sampled at the junction of the tumor and adjacent liver tissues in a 1:1 ratio at the 12 (A), 3 (B), 6 (C), and 9 (D) o'clock positions, and at least one tissue was sampled at the tumor center (E), the liver tissue ≤1 cm away from the tumor (F), and the liver tissue >1 cm away from the tumor (G). Surgical specimens were embedded in paraffin, and 5‐μm‐thick sections were cut from each block and stained with hematoxylin and eosin for histological examination.

### Clinicopathological characteristics and definitions

We collected data from serological examinations carried out within 2 weeks before surgery. Pathological diagnosis was made by two pathologists based on histology. MiVI was defined as the presence of tumor cell nests in the portal vein, hepatic vein, or capsular vessel lined by endothelium, visible only under microscopy. The two‐tiered MiVI grading scheme classified the specimens into the MiVI‐positive and MiVI‐negative groups. The MiVI‐TTG scheme classified the specimens as M0 (no MiVI)(Figure 2b), M1 (1–5 sites of MiVI occurring in the tumor‐adjacent liver tissue ≤1 cm away from the tumor capsule)(Figure 2C), and M2 (>5 MiVI sites, or any MiVI existing in the distant liver tissue >1 cm away from the tumor capsule) (Figure 2D). MaVI was defined as the invasion of the first‐ and second‐order branches of the portal veins, hepatic arteries or hepatic veins. Satellite nodules (SN) were defined as tumor cell nests present on microscopy or tumors with a maximum diameter of <2 cm resenting within 2 cm of the main tumor on macroscopy. Tumor stage was defined according to the 8th edition of the American Joint Committee Cancer staging manual.

### Follow‐up

All enrolled patients were regularly followed‐up after discharge. The frequency of follow‐up was scheduled to be every 3 months during the first 2 years and every 6–12 months thereafter. Routine examinations at the follow‐up visits included testing serum tumor markers and contrast‐enhanced abdominal magnetic resonance imaging. Patients with recurrence of cHCC were provided with further antitumor treatments, including TACE, radiofrequency ablation, radiotherapy, or liver resection. Recurrence‐free survival (RFS) was defined as the time interval from surgery to the detection of recurrence or latest follow‐up. Early recurrence (ER) was defined as recurrence within 2 years after surgery. Overall survival (OS) was defined as the time interval from surgery to either the latest follow‐up visit or death from any cause. The collection of survival information was completed on October 31, 2021.

### Statistical analysis

Categorical variables were presented as numbers (percentages) and compared using the chi‐square test or Fisher's exact test. Normally distributed continuous variables were presented as mean (standard deviation [IQR]) and compared using the Mann–Whitney U test. Statistical significance was set at *p* < 0.05. R (version 4.1.0, packages “rms,” “timeROC,” “ggDCA,” “survminer” and “survival”) was used to perform the statistical analysis.

The optimal thresholds were determined by analyzing the ROC curves and the Youden index. Variables with *p* < 0.05 in univariable regression analysis were further employed for multivariable regression analysis via the stepwise backward likelihood ratio method. Kaplan–Meier curves and log‐rank tests were used to measure the differences between various subgroups. Harrell's c‐index and time‐dependent areas under the receiver operating characteristic curve (tdAUC) were employed to evaluate the discriminative ability. Decision curve analysis (DCA) was conducted to estimate clinical net benefits using the bootstrapping resampling method (*n* = 1000).^25^

## RESULTS

### Baseline features and risk factors for MiVI

Overall, 208 patients who met the criteria were divided into three groups as M0 (38.9%), M1 (36.5%), and M2 (24.5%) group based on MiVI‐TTG. A comparison of the baseline characteristics between the groups is shown in Table 1. Variables, including tumor size ≥5 cm, the presence of MaVI and SN, Edmondson‐Steiner classification III/IV, and AFP≥400 ng/ml, were significantly different between the M0 and M2 groups. Among the four variables, only SN, was significantly different between the M1 and M2 groups. No variables differed between the M0 and M1 groups.

**TABLE 1:** Baseline characteristics based on three‐tiered MiVI grading system

Variables	MiVI three‐tiered grading			*p*‐value			
	M0 (*n* = 81)	M1 (*n* = 76)	M2 (*n* = 51)	M0VSM1	M0VSM2	M1VSM2	Total
Gender (male), *n* (%)	72 (88.9%)	65 (85.5%)	40 (78.4%)	0.695	0.167	0.426	0.257
Age (> = 65 years), *n* (%)	14 (17.3%)	16 (21.1%)	8 (15.7%)	0.714	0.691	1	0.599
HBV (yes), *n* (%)	67 (82.7%)	64 (84.2%)	43 (84.3%)	0.971	1	1	0.958
HCV (yes), *n* (%)	1 (1.2%)	1 (1.3%)	1 (2.0%)	1	1	1	0.937
Cirrhosis (yes), *n* (%)	57 (70.4%)	44 (57.9%)	36 (70.6%)	0.143	1	0.206	0.184
Tumor number (multiple), *n* (%)	24 (29.6%)	17 (22.4%)	16 (31.4%)	0.393	0.986	0.353	0.455
Tumor size (> = 5 cm), *n* (%)	36 (44.4%)	41 (53.9%)	36 (70.6%)	0.303	0.006	0.090	0.013
Tumor capsule (incomplete), *n* (%)	45 (55.6%)	46 (60.5%)	24 (47.1%)	0.639	0.44	0.189	0.326
Macrovascular invasion (present), *n* (%)	14 (17.3%)	15 (19.7%)	20 (39.2%)	0.849	0.009	0.027	0.009
Lymph node metastasis (present), *n* (%)	9 (11.1%)	10 (13.2%)	6 (11.8%)	0.882	1	1	0.923
Satellite nodules (present), *n* (%)	21 (25.9%)	22 (28.9%)	32 (62.7%)	0.806	<0.001	<0.001	<0.001
Edmondson‐Steiner classification (III/IV), *n* (%)	28 (34.6%)	30 (39.5%)	31 (60.8%)	0.638	0.006	0.030	0.009
RBC (> = 4*10^12^/L), *n* (%)	9 (11.1%)	7 (9.2%)	3 (5.9%)	0.897	0.48	0.729	0.597
Hb (<120*g/L), *n* (%)	7 (8.6%)	4 (5.3%)	3 (5.9%)	0.606	0.806	1	0.674
PLT (<100*10^9^/L), *n* (%)	11 (13.6%)	9 (11.8%)	5 (9.8%)	0.931	0.709	0.944	0.808
ALT (> = 40 U/L), *n* (%)	25 (30.9%)	22 (28.9%)	18 (35.3%)	0.93	0.735	0.575	0.748
AST (> = 40 U/L), *n* (%)	26 (32.1%)	36 (47.4%)	22 (43.1%)	0.073	0.272	0.774	0.135
PT (> = 13 s), *n* (%)	30 (37.0%)	26 (34.2%)	26 (51.0%)	0.839	0.162	0.089	0.142
TB (> = 17.1 umol/L), *n* (%)	15 (18.5%)	20 (26.3%)	18 (35.3%)	0.326	0.050	0.376	0.096
ALB (<40 g/L), *n* (%)	29 (35.8%)	20 (26.3%)	19 (37.3%)	0.267	1	0.265	0.326
ALP (> = 100 U/L), *n* (%)	28 (34.6%)	25 (32.9%)	24 (47.1%)	0.958	0.212	0.155	0.227
AFP (> = 400 ng/mL), *n* (%)	13 (16.0%)	22 (28.9%)	22 (43.1%)	0.080	0.001	0.145	0.003
CEA (> = 5 ng/ml), *n* (%)	7 (8.6%)	8 (10.5%)	11 (21.6%)	0.897	0.0648	0.145	0.074
CA19‐9 (> = 40 U/ml), *n* (%)	20 (24.7%)	24 (31.6%)	21 (41.2%)	0.434	0.0719	0.358	0.138
Resection type							
Minor resection, *n* (%)	28 (34.6%)	32 (42.1%)	13 (25.5%)	0.42	0.366	0.084	0.156
Major resection, *n* (%)	53 (65.4%)	44 (57.9%)	38 (74.5%)				
Lymph node dissection (yes), *n* (%)	12 (14.8%)	14 (18.4%)	9 (17.6%)	0.695	0.85	1	0.82
Intraoperative blood loss (> = 200 ml), *n* (%)	52 (64.2%)	45 (59.2%)	30 (58.8%)	0.632	0.663	1	0.759
Intraoperative blood transfusion (yes), *n* (%)	16 (19.8%)	11 (14.5%)	13 (25.5%)	0.506	0.576	0.186	0.3
Child‐Pugh							
A, *n* (%)	79 (97.5%)	73 (96.1%)	47 (92.2%)	0.942	0.31	0.585	0.329
B, *n* (%)	2 (2.5%)	3 (3.9%)	4 (7.8%)				
AJCC 8th							
IA‐II, *n* (%)	65 (80.2%)	59 (77.6%)	34 (66.7%)	0.837	0.122	0.245	0.188
III‐IV, *n* (%)	16 (19.8%)	17 (22.4%)	17 (33.3%)				

Abbreviations: AFP, alpha‐fetoprotein; AJCC, American Joint Committee on Cancer; ALB, albumin; ALP, alkaline phosphatase; ALT, alanine aminotransferase; AST, aspartate aminotransferase; CA19‐9, carbohydrate antigen 19–9; CEA, carcinoembryonic antigen; Hb, hemoglobin; HBV, hepatitis B virus; HCV, hepatitis C virus; MiVI, microvascular invasion; PLT, platelet count; PT, prothrombin time; RBC, red blood cell; TB, total bilirubin.

Similarly, univariable logistic regression analysis revealed that the presence of MiVI was related to tumor size ≥5 cm and the presence of SN and AFP ≥400 ng/ml (Table S1), while multivariate logistic regression analysis only identified AFP ≥400 ng/ml as an independent risk factor for MiVI (Table 2). In our research on MiVI‐TTG, we explored the risk factors for M2 in the MiVI‐positive population. Univariable logistic regression analysis indicated that the presence of SN, MaVI, and Edmondson–Steiner classification III/IV are risk factors for M2 (Table S1). Multivariate logistic regression analysis identified SN as an independent risk factor for M2 (Table 2).

**TABLE 2:** Multivariable logistics regression analysis for MiVI two‐tiered grading and MiVI three‐tiered grading

Variables	MiVI two‐tiered grading		MiVI three‐tiered grading	
	OR (95% CI)	*p*‐value	OR (95% CI)	*p*‐value
Tumor size (> = 5 cm)	1.5 (0.82–2.72)	0.186		
Macrovascular invasion (present)			1.91 (0.81–4.53)	0.142
Satellite nodules (present)	1.85 (0.99–3.46)	0.055	3.66 (1.68–7.94)	0.001
Edmondson‐Steiner classification (III/IV)			1.95 (0.89–4.27)	0.095
AFP (> = 400 ng/mL)	2.28 (1.1–4.72)	0.026		

Abbreviations: AFP, alpha‐fetoprotein; CI, confidence interval; MiVI, microvascular invasion; OR, odds ratio.

### Comparison of predictive performance

**FIGURE 3:**
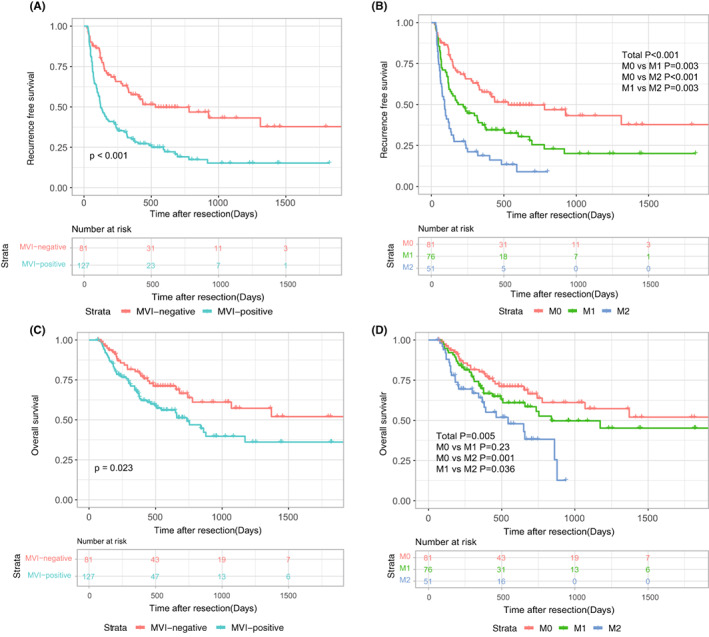
Kaplan–Meier survival curves of RFS based on MiVI two‐tiered MiVI scheme (A) and MiVI three‐tiered MiVI scheme (B); Kaplan–Meier survival curves of OS based on MiVI two‐tiered MiVI scheme (C) and MiVI three‐tiered MiVI scheme (D).

In this research, the median follow‐up time for OS and RFS was 16.0 and 8.8 months, respectively. In the two‐tiered MiVI scheme, the 1‐, 2‐, and 3‐year RFS rates for MiVI‐positive group were 30.0%, 17.4%, and 15.2%, comparing with those for MiVI‐negative group being 57.6%, 49.7% and 43.2%(Figure 3A). Additionally, the 1‐, 2‐, and 3‐year OS rates for MiVI‐positive group were 67.3%, 51.2%, and 39.7%, comparing with those for MiVI‐negative group being 80.2%, 66.5% and 57.3%(Figure 3C). Survival curves were widely separated in the Kaplan–Meier analysis for OS and RFS (both *p* < 0.05).

In the MiVI‐TTG scheme, the 1‐, 2‐, and 3‐year RFS rates for M0 group were 57.6%, 49.7%, and 43.2%, comparing to those for M1 group being 37.5%, 25.5%, and 20.1%, comparing with the 1‐ and 2‐year RFS rates for M2 group being 18.8% and 8.9% (Figure 3B). Kaplan–Meier analysis showed that the survival curves were separated from each other (*p* < 0.05) (Figure 3B).

Additionally, the 1‐, 2‐, and 3‐year OS rates for M0 group were 80.2%, 66.5%, and 57.3%, comparing with those for M1 group being 69.8%, 58.5%, and 49.7%, and those for M2 group being 64.1%, 38.3%, and 12.8% (Figure 3D). According to the log‐rank test, the survival curves of M0 and M1 group were not significantly different (*p* = 0.23), but the survival curves of M0 and M1 group were significantly different from those of M2 group (*p* < 0.05, Figure 3D).

Cox regression analyses showed that CA19‐9 ≥ 40 U/ml and the presence of MiVI, M1, M2, and MaVI were independent risk factors for ER (Table 3). The presence of MiVI, together with CA19‐9 ≥ 40 U/ml and incomplete tumor capsule, comprised the independent risk factors for OS, while M2, together with incomplete tumor capsule alone, was independent risk factors for OS (Table S2).

**TABLE 3:** Univariable and multivariable cox regression analysis for early recurrence

Variables	Univariable cox analysis		Multivariable cox analysis based on two‐tiered MiVI scheme		Multivariable cox analysis based on three‐tiered MiVI scheme	
	OR (95% CI)	*p*‐value	OR (95% CI)	*p*‐value	OR (95% CI)	*p*‐value
Gender (male)	0.72 (0.46–1.13)	0.15				
Age (> = 65 years)	0.91 (0.58–1.42)	0.669				
HBV (yes)	1.05 (0.65–1.69)	0.845				
HCV (yes)	1.51 (0.37–6.12)	0.562				
Cirrhosis (yes)	0.96 (0.67–1.36)	0.808				
Tumor number (multiple)	1.09 (0.74–1.59)	0.67				
Tumor size (> = 5 cm)	1.71 (1.21–2.41)	0.003	1.02 (0.68–1.54)	0.921	0.99 (0.66–1.5)	0.975
Tumor capsule (incomplete)	0.93 (0.66–1.31)	0.683				
Microvascular invasion (present)	2.32 (1.59–3.38)	<0.001	2.09 (1.41–3.1)	<0.001		
Microvascular invasion three‐tiered grading						
M1 vs M0	1.85 (1.22–2.81)	0.004			1.91 (1.24–2.95)	0.003
M2 vs M0	3.41 (2.19–5.29)	<0.001			2.45 (1.5–4.01)	<0.001
Macrovascular invasion (present)	2.18 (1.5–3.16)	<0.001	2.08 (1.34–3.21)	0.001	1.99 (1.28–3.1)	0.002
Lymph node metastasis (present)	1.71 (1.05–2.79)	0.031	1.24 (0.59–2.64)	0.57	1.28 (0.61–2.71)	0.515
Satellite nodules (present)	1.85 (1.31–2.6)	<0.001	1.33 (0.92–1.93)	0.132	1.25 (0.84–1.85)	0.275
Edmondson‐Steiner classification (III/IV)	1.13 (0.8–1.59)	0.497				
RBC (> = 4 × 10^12^/L)	0.99 (0.55–1.79)	0.968				
Hb (<120 × g/L)	1.23 (0.65–2.35)	0.525				
PLT (<100 × 10^9^/L)	0.77 (0.43–1.37)	0.372				
ALT (> = 40 U/L)	0.95 (0.66–1.36)	0.762				
AST (> = 40 U/L)	0.79 (0.56–1.13)	0.197				
PT (> = 13 s)	1.18 (0.83–1.66)	0.353				
TB (> = 17.1 μmol/L)	0.94 (0.64–1.39)	0.751				
ALB (<40 g/L)	1.06 (0.74–1.53)	0.735				
ALP (> = 100 U/L)	1.46 (1.03–2.06)	0.032	0.98 (0.66–1.44)	0.904	0.96 (0.65–1.42)	0.828
AFP (> = 400 ng/ml)	1.95 (1.36–2.79)	<0.001	1.34 (0.89–2)	0.159	1.31 (0.87–1.97)	0.198
CEA (> = 5 ng/ml)	1.17 (0.71–1.93)	0.526				
CA19‐9 (> = 40 U/ml)	2.05 (1.45–2.91)	<0.001	1.81 (1.22–2.69)	0.003	1.82 (1.22–2.7)	0.003
Resection type (major resection)	1.54 (1.07–2.23)	0.021	1.26 (0.82–1.91)	0.287	1.25 (0.82–1.9)	0.297
Lymph node dissection (yes)	1.75 (1.14–2.67)	0.01	0.9 (0.45–1.78)	0.757	0.94 (0.47–1.86)	0.853
Intraoperative blood loss (> = 200 ml)	1.25 (0.88–1.78)	0.214				
Intraoperative blood transfusion (yes)	1.14 (0.75–1.74)	0.546				

Abbreviations: AFP, alpha‐fetoprotein; AJCC, American Joint Committee on Cancer; ALB, albumin; ALP, alkaline phosphatase; AST, aspartate aminotransferase; CA19‐9, carbohydrate antigen 19‐9; CEA, carcinoembryonic antigen; CI, confidence interval; DCP, decarboxylic prothrombin; GGT, gamma‐glutamyl transpeptidase; Hb, hemoglobin; HBV, hepatitis B virus; HCV, hepatitis C virus; MiVI, microvascular invasion; NLR, neutrophils/lymphocytes ratio; OR, odds ratio; PLT, platelet count; PT, prothrombin time; RBC, red blood cell; TB, total bilirubin.

In predicting ER, the C‐index of MiVI‐TTG (0.6278,95% CI 0.5908–0.6692) was significantly higher than that of the two‐tiered MiVI scheme (0.5977,95% CI 0.5608–0.6392) according to P index of 0.006. In predicting OS, there was no statistical difference in the the C‐index of MiVI‐TTG (0.5882, 95%CI 0.5312–0.6488) and the two‐tiered MiVI scheme (0.5634, 95% CI 0.5012–0.6188, *p* = 0.086).

**FIGURE 4:**
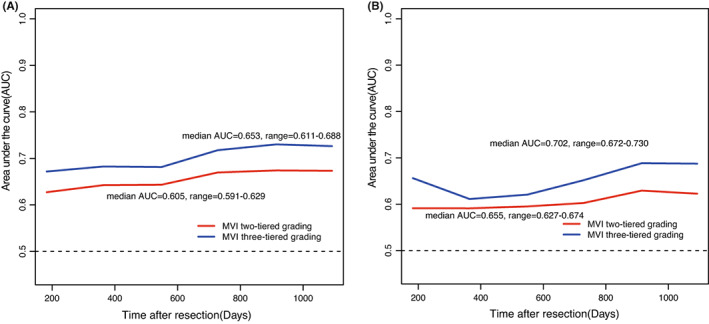
TdROC curves for MiVI two‐tiered MiVI scheme and MiVI three‐tiered MiVI scheme in the prediction of early recurrence (A) and OS (B).

**FIGURE 5:**
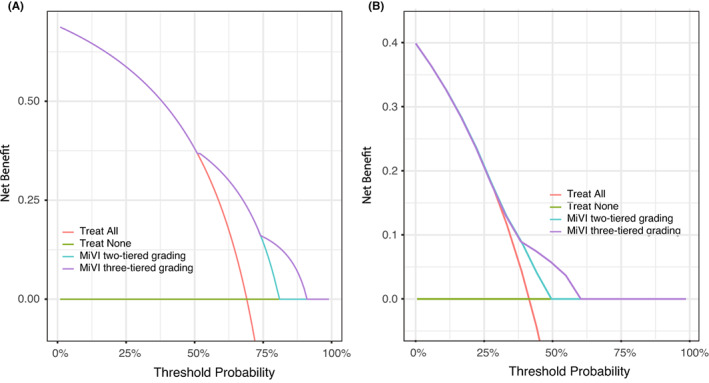
Decision curve analysis on MiVI two‐tiered MiVI scheme and MiVI three‐tiered MiVI scheme for the prediction of early recurrence (A) and OS (B).

According to tdAUC analysis, MiVI‐TTG had better discrimination than the two‐tiered MiVI scheme in predicting ER and OS (Figure 4). In predicting ER, median tdAUC values of the MiVI‐TTG and two‐tiered MiVI schemes were 0.702 (range, 0.672–0.730) and 0.655 (range, 0.627–0.674), respectively. In predicting OS, Median tdAUC values of the MiVI‐TTG and two‐tiered MiVI schemes were 0.653 (range, 0.611–0.688) and 0.605 (range, 0.591–0.629), respectively. DCA revealed that the MiVI‐TTG scheme provided better net benefits than the two‐tiered MiVI scheme (Figure 5).

## DISCUSSION

Patients with cHCC have a dismal prognosis even after hepatic resection or liver transplantation, because of the high recurrence rate. However, the management of cHCC remains unclear, because of a poor understanding of its pathological features and prognosis. CHCC and ICC share the same staging system in the 8th edition of the AJCC staging manual.^5^ In the guidelines for the treatment and management of PLC, the treatment strategies for cHCC have not been described individually or in detail.^26^, ^27^, ^28^, ^29^ Research on the pathological features of cHCC would be of great help in elucidating the aggressive biological behaviors of tumor and exploring the mechanism of recurrence, thus predicting the prognosis after surgery and providing individual treatment strategies, such as appropriate resection types and anti‐recurrence adjuvant treatments such as TACE.

To date, multiple types of classifications and definitions have been proposed.^9^, ^17^, ^18^, ^19^, ^20^, ^21^, ^22^, ^23^ Among them, a definition (MiVI is defined as the presence of tumor cell nests in the portal vein, hepatic vein, or tumor capsular vessel lined by endothelium, which were visible only under microscopy.) and a classification of two‐tiered MiVI grading schemes (i.e., present or absent) has been widely accepted and applied by most researchers.^9^, ^17^, ^22^, ^24^ The two‐tiered grading scheme is simple and ignores the significant impact of the number and distance of MiVI on the prognosis.^18^, ^21^ Accordingly, a three‐tiered grading scheme of MiVI (MiVI‐TTG) comprising the number and distance of MiVI was proposed as a practice guideline for the pathological diagnosis of primary liver cancer by LCPGC.^17^ A large multicenter study showed that MiVI‐TTG has a greater prognostic value than the two‐tiered MiVI scheme in patients with HCC after surgery and can be easily implemented in multiple centers.^22^ Compared with some other classifications involving special immunohistochemistry or more complicated grading, MiVI‐TTG appears to save meaningless time, cost, and pathologist workload in clinical practice. Nevertheless, whether the prognostic value of MiVI‐TTG is greater than that of the two‐tiered scheme in patients with cHCC after surgery has not been verified.

In this research, the proportions of M0, M1, and M2 were 38.9%, 36.5%, and 24.5%. MiVI‐positive patients, comprising patients with M1 and M2, accounted for 61.1% of all patients enrolled. According to the previous researches, the incidence rate of MiVI in cHCC ranges from 33% to 68.7%.^4^, ^6^, ^11^, ^12^, ^13^, ^14^, ^15^ Consistent with previous studies, the incidence of MiVI reported in our center was relatively high. The incidence of MiVI is affected by the diagnostic criteria applied among different centers and whether appropriate and careful sampling procedures have been performed.^30^ This study only recruited patients who underwent hepatic surgery at our center after 2015. Our center has strictly followed the 7‐point sample collection protocol and detailed definition of MiVI described in “Practice guidelines for the pathological diagnosis of primary liver cancer: 2015 update”^17^ proposed by LCPGC since 2015, which resulted in a higher MiVI detection rate than other centers following the original 3‐point sampling protocol proposed by Torbenson and varied definitions of MiVI.^19^, ^20^, ^21^, ^23^ Additionally, the reported detection rate of MiVI increases gradually from year to year as more attention is being paid to MiVI and detection technologies improve.^4^, ^6^, ^11^, ^12^, ^13^, ^14^, ^15^, ^30^

The present study indicated that cHCC with severe MiVI status had a dismal prognosis, with significantly worse RFS and OS. Previous studies have shown that adjuvant treatments, such as TACE, improve the outcomes of patients with ICC or HCC.^31^, ^32^, ^33^, ^34^ Given that cHCC consists of HCC and ICC,^1^, ^3^ further research should be conducted to clarify whether adjuvant treatments are beneficial to cHCC patients with MiVI, especially for those with M2 classification. In our research, MiVI‐TTG showed more prognostic value in predicting OS and RFS than the two‐tiered MiVI scheme with larger c‐index, tdAUC, and net benefit rate. MiVI‐TTG contributes to the exploration of the relationship between vascular invasion and prognosis, and thus promotes the construction of individual cHCC staging systems in the AJCC staging manual.

Given that MiVI has a significant impact on recurrence and survival after hepatic resection, we aimed to explore the risk factors for MiVI in cHCC. The risk factors of MiVI include the presence of macrovascular invasion (MaVI) and portal hypertension, incomplete tumor capsule, and AFP ≥ 400 ng/ml , as has been reported in different studies on cHCC.^4^, ^12^, ^13^ In this study, multivariable logistic regression analysis showed AFP≥400 ng/ml was an independent risk factor for MiVI‐positive and the presence of SN for M2. Regarding to this, previous studies have shown that AFP and MiVI were both associated with aggressive tumor behaviors in both ICC and HCC,^8^, ^9^ and HCC can arise from satellite nodules which are that result from MiVI.^18^

Our study had several limitations. First, this research was based on a limited sample size and retrospective data; therefore, information bias and heterogeneity in clinicopathological features should be considered. Second, all the enrolled patients were from China. The detection rate of MiVI is relatively high compared with other centers overseas,^9^, ^10^ because the specimens proceeded strictly following the 7‐point sample collection protocol. The definition of MiVI in our research is different from that in other researches.^19^, ^20^, ^21^ Moreover, there were no clinical data from other centers to validate the value of MiVI‐TTG. Therefore, the generality of these conclusions may be limited. Third, the Cox and logistic regression analyses employed in this research were antiquated, and some up‐to‐date methods, such as least absolute shrinkage and selection operator analysis, random forest, and extreme gradient boosting, may have improved the accuracy of this research.

## CONCLUSIONS

In conclusion, our study shows that cHCC patients with severe MiVI have a dismal prognosis with significantly worse RFS and OS after hepatic resection. AFP≥400 ng/ml was the independent risk factor for MiVI, and satellite nodules were independent risk factors for M2. MiVI‐TTG has a greater prognostic value in predicting OS and RFS than the traditional two‐tiered MiVI pathological scheme, with a larger c‐index, tdAUC, and net benefit rate in patients with cHCC.

## AUTHOR CONTRIBUTIONS

YW, YZ, and JL contributed to the design of the study. YW, HL, YC, QH, and JZ contributed to data collection and interpretation or analysis. YW wrote the manuscript. All authors approved the final manuscript.

## FUNDING INFORMATION

This research was supported by Startup Fund for Scientific Research, Fujian Medical University (2019QH1294), Startup Fund for Scientific Research, Fujian Medical University (2019QH1297), Health research talent training project of Fujian province (2019‐1‐85), Fuzhou Science and Technology Bureau project (2020‐WS‐92), Fuzhou Science and Technology Bureau project (2020‐WS‐57), Provincial Clinical Research Center for Hepatobiliary and Pancreatic Tumors (2020Y2013), Key Clinical Specialty Discipline Construction Program of Fuzhou (201912002), and the Scientific Foundation of Fuzhou Municipal Health commission (2021‐S‐wp1).

## CONFLICT OF INTEREST

The authors have no conflicts of interest to report.

## ETHICAL APPROVAL STATEMENT

Our research was conducted to the ethical guideline of the 1975 Declaration of Helsinki and obtained approval from the Institutional Ethics Committee of Fujian Medical University Affiliated Mengchao Hepatobiliary Hospital. Informed consent has been obtained from patients participating in this research.

## Supporting information


Table S1
Table S2

## Data Availability

The data that support the findings of this study are available from the corresponding author upon reasonable request.

## References

[1] Lee J‐H , Chung GE , Yu SJ , et al. Long‐term prognosis of combined hepatocellular and cholangiocarcinoma after curative resection comparison with hepatocellular carcinoma and cholangiocarcinoma. J Clin Gastroenterol. 2011;45(1):69‐75. 20142755 10.1097/MCG.0b013e3181ce5dfa

[2] Yamashita Y‐I , Aishima S , Nakao Y , et al. Clinicopathological characteristics of combined hepatocellular cholangiocarcinoma from the viewpoint of patient prognosis after hepatic resection: high rate of early recurrence and its predictors. Hepatol Res. 2020;50(7):863‐870. 32335986 10.1111/hepr.13507

[3] Yin X , Zhang B‐H , Qiu S‐J , et al. Combined hepatocellular carcinoma and cholangiocarcinoma: clinical features, treatment modalities, and prognosis. Ann Surg Oncol. 2012;19(9):2869‐2876. 22451237 10.1245/s10434-012-2328-0

[4] Wang T , Yang X , Tang H , et al. Integrated nomograms to predict overall survival and recurrence‐free survival in patients with combined hepatocellular cholangiocarcinoma (cHCC) after liver resection. Aging. 2020;12(15):15334‐15358. 32788423 10.18632/aging.103577 PMC7467372

[5] Chun YS , Pawlik TM , Vauthey J‐N . 8th edition of the AJCC cancer staging manual: pancreas and hepatobiliary cancers. Ann Surg Oncol. 2018;25(4):845‐847. 28752469 10.1245/s10434-017-6025-x

[6] Tian M‐X , Luo L‐P , Liu W‐R , et al. Development and validation of a prognostic score predicting recurrence in resected combined hepatocellular cholangiocarcinoma. Cancer Manag Res. 2019;11:5187‐5195. 31239773 10.2147/CMAR.S195964 PMC6556465

[7] Hu L‐S , Weiss M , Popescu I , et al. Impact of microvascular invasion on clinical outcomes after curative‐intent resection for intrahepatic cholangiocarcinoma. J Surg Oncol. 2019;119(1):21‐29. 30466151 10.1002/jso.25305

[8] Tang Z , Liu W‐R , Zhou P‐Y , et al. Prognostic value and predication model of microvascular invasion in patients with intrahepatic cholangiocarcinoma. J Cancer. 2019;10(22):5575‐5584. 31632502 10.7150/jca.32199 PMC6775679

[9] Chan AWH , Zhong J , Berhane S , et al. Development of pre and post‐operative models to predict early recurrence of hepatocellular carcinoma after surgical resection. J Hepatol. 2018;69(6):1284‐1293. 30236834 10.1016/j.jhep.2018.08.027

[10] Shindoh J , Kobayashi Y , Kawamura Y , et al. Microvascular invasion and a size cutoff value of 2 cm predict long‐term oncological outcome in multiple hepatocellular carcinoma: reappraisal of the American joint committee on cancer staging system and validation using the surveillance, epidemiology, and end‐results database. Liver Cancer. 2020;9(2):156‐166. 32399430 10.1159/000504193 PMC7206607

[11] Chu K‐j , Lu C‐d , Dong H , Fu X‐h , Zhang H‐w , Yao X‐p . Hepatitis B virus‐related combined hepatocellular‐cholangiocarcinoma: clinicopathological and prognostic analysis of 390 cases. Eur J Gastroenterol Hepatol. 2014;26(2):192‐199. 24370644 10.1097/MEG.0b013e3283625df9

[12] Wang X , Wang W , Ma X , et al. Combined hepatocellular‐cholangiocarcinoma: which preoperative clinical data and conventional MRI characteristics have value for the prediction of microvascular invasion and clinical significance? Eur Radiol. 2020;30(10):5337‐5347. 32385649 10.1007/s00330-020-06861-2 PMC7476977

[13] Wang Y , Zhou C‐W , Zhu G‐Q , et al. A multidimensional nomogram combining imaging features and clinical factors to predict the invasiveness and metastasis of combined hepatocellular cholangiocarcinoma. Ann Transl Med. 2021;9(20):1518. 34790724 10.21037/atm-21-2500 PMC8576707

[14] Zhang H , Yu X , Xu J , Li J , Zhou Y . Combined hepatocellular‐cholangiocarcinoma: an analysis of clinicopathological characteristics after surgery. Medicine (Baltimore). 2019;98(38):e17102. 31567946 10.1097/MD.0000000000017102 PMC6756736

[15] Zhang J , Wang X , Zhang L , et al. Radiomics predict postoperative survival of patients with primary liver cancer with different pathological types. Ann Transl Med. 2020;8(13):820. 32793665 10.21037/atm-19-4668 PMC7396247

[16] Lee S , Kang TW , Song KD , et al. Effect of microvascular invasion risk on early recurrence of hepatocellular carcinoma after surgery and radiofrequency ablation. Ann Surg. 2021;273(3):564‐571. 31058694 10.1097/SLA.0000000000003268

[17] Cong W‐M , Bu H , Chen J , et al. Practice guidelines for the pathological diagnosis of primary liver cancer: 2015 update. World J Gastroenterol. 2016;22(42):9279‐9287. 27895416 10.3748/wjg.v22.i42.9279 PMC5107692

[18] Feng L‐H , Dong H , Lau W‐Y , et al. Novel microvascular invasion‐based prognostic nomograms to predict survival outcomes in patients after R0 resection for hepatocellular carcinoma. J Cancer Res Clin Oncol. 2017;143(2):293‐303. 27743138 10.1007/s00432-016-2286-1 PMC11819416

[19] Fujita N , Aishima S , Iguchi T , et al. Histologic classification of microscopic portal venous invasion to predict prognosis in hepatocellular carcinoma. Hum Pathol. 2011;42(10):1531‐1538. 21496875 10.1016/j.humpath.2010.12.016

[20] Hidaka M , Eguchi S , Okuda K , et al. Impact of anatomical resection for hepatocellular carcinoma with microportal invasion (vp1): a multi‐institutional study by the Kyushu study Group of Liver Surgery. Ann Surg. 2020;271(2):339‐346. 30048313 10.1097/SLA.0000000000002981

[21] Roayaie S , Blume IN , Thung SN , et al. A system of classifying microvascular invasion to predict outcome after resection in patients with hepatocellular carcinoma. Gastroenterology. 2009;137(3):850‐855. 19524573 10.1053/j.gastro.2009.06.003 PMC2739450

[22] Sheng X , Ji Y , Ren G‐P , et al. A standardized pathological proposal for evaluating microvascular invasion of hepatocellular carcinoma: a multicenter study by LCPGC. Hepatol Int. 2020;14(6):1034‐1047. 33369707 10.1007/s12072-020-10111-4

[23] Sumie S , Nakashima O , Okuda K , et al. The significance of classifying microvascular invasion in patients with hepatocellular carcinoma. Ann Surg Oncol. 2014;21(3):1002‐1009. 24254204 10.1245/s10434-013-3376-9

[24] Erstad DJ , Tanabe KK . Prognostic and therapeutic implications of microvascular invasion in hepatocellular carcinoma. Ann Surg Oncol. 2019;26(5):1474‐1493. 30788629 10.1245/s10434-019-07227-9

[25] Vickers AJ , Elkin EB . Decision curve analysis: a novel method for evaluating prediction models. Med Decis Making. 2006;26(6):565‐574. 17099194 10.1177/0272989X06295361 PMC2577036

[26] EASL Clinical Practice Guidelines . Management of hepatocellular carcinoma. J Hepatol. 2018;69(1):182‐236. 29628281 10.1016/j.jhep.2018.03.019

[27] Heimbach JK , Kulik LM , Finn RS , et al. AASLD guidelines for the treatment of hepatocellular carcinoma. Hepatology. 2018;67(1):358‐380. 28130846 10.1002/hep.29086

[28] Kudo M , Matsui O , Izumi N , et al. JSH consensus‐based clinical practice guidelines for the Management of Hepatocellular Carcinoma: 2014 update by the liver cancer study Group of Japan. Liver Cancer. 2014;3(3–4):458‐468. 26280007 10.1159/000343875 PMC4531423

[29] Omata M , Cheng A‐L , Kokudo N , et al. Asia‐Pacific clinical practice guidelines on the management of hepatocellular carcinoma: a 2017 update. Hepatol Int. 2017;11(4):317‐370. 28620797 10.1007/s12072-017-9799-9 PMC5491694

[30] Zhang X , Li J , Shen F , Lau WY . Significance of presence of microvascular invasion in specimens obtained after surgical treatment of hepatocellular carcinoma. J Gastroenterol Hepatol. 2018;33(2):347‐354. 28589639 10.1111/jgh.13843

[31] Cheng Z , Lei Z , Jin X , et al. Postoperative adjuvant transarterial chemoembolization for intrahepatic cholangiocarcinoma patients with microvascular invasion: a propensity score analysis. J Gastrointest Oncol. 2021;12(2):819‐830. 34012669 10.21037/jgo-20-443 PMC8107590

[32] Li Z , Lei Z , Xia Y , et al. Association of Preoperative Antiviral Treatment with Incidences of microvascular invasion and early tumor recurrence in hepatitis B virus‐related hepatocellular carcinoma. JAMA Surg. 2018;153(10):e182721. 30073257 10.1001/jamasurg.2018.2721 PMC6233791

[33] Wang H , Du P‐C , Wu M‐C , Cong W‐M . Postoperative adjuvant transarterial chemoembolization for multinodular hepatocellular carcinoma within the Barcelona clinic liver cancer early stage and microvascular invasion. Hepatobiliary Surg Nutr. 2018;7(6):418‐428. 30652086 10.21037/hbsn.2018.09.05 PMC6295398

[34] Wang H , Yu H , Qian Y‐W , Cao Z‐Y , Wu M‐C , Cong W‐M . Impact of surgical margin on the prognosis of early hepatocellular carcinoma (≤5 cm): a propensity score matching analysis. Front Med (Lausanne). 2020;7:139. 32478080 10.3389/fmed.2020.00139 PMC7232563

