# Supplementary material for: Prognostic significance of three‐tiered pathological classification for microvascular invasion in patients with combined hepatocellular‐cholangiocarcinoma following hepatic resection
Source: Cancer Med. 2022 Nov 10;12(5):5233–44. doi: 10.1002/cam4.5328 (PMC10028161; doi:10.1002/cam4.5328)
Supplement: Supplementary file 1 — Table S1 Table S2 [file CAM4-12-5233-s001.docx]

Supplementary tables

Table S1: Univariable logistics regression analysis for MiVI two-tiered grading and MiVI three-tiered grading.

| Variables | MiVI two-tiered grading | | MiVI three-tiered grading | |  |
| --- | --- | --- | --- | --- | --- |
|  | OR (95% CI) | P-value | OR (95% CI) | P-value | |
| Gender(male) | 0.6(0.26-1.37) | 0.223 | 0.6(0.26-1.37) | 0.223 | |
| Age(>=65 years) | 1.12(0.54-2.31) | 0.769 | 1.12(0.54-2.31) | 0.769 | |
| HBV(yes) | 1.12(0.53-2.36) | 0.770 | 1.12(0.53-2.36) | 0.770 | |
| HCV(yes) | 1.28(0.11-14.35) | 0.841 | 1.28(0.11-14.35) | 0.841 | |
| Cirrhosis(yes) | 0.72(0.39-1.3) | 0.275 | 0.72(0.39-1.3) | 0.275 | |
| Tumor number(multiple) | 0.83(0.45-1.55) | 0.566 | 0.83(0.45-1.55) | 0.566 | |
| Tumor size (>=5cm) | 1.92(1.09-3.39) | 0.023 | 1.92(1.09-3.39) | 0.023 | |
| Tumor capsule(incomplete) | 0.98(0.56-1.72) | 0.951 | 0.98(0.56-1.72) | 0.951 | |
| Macrovascular invasion(present) | 1.82(0.91-3.65) | 0.091 | 1.82(0.91-3.65) | 0.091 | |
| Lymph node metastasis (present) | 1.15(0.48-2.75) | 0.748 | 1.15(0.48-2.75) | 0.748 | |
| Satellite nodules(present) | 2.11(1.15-3.89) | 0.016 | 2.11(1.15-3.89) | 0.016 | |
| Edmondson-Steiner classification(III/IV) | 1.75(0.98-3.11) | 0.057 | 1.75(0.98-3.11) | 0.057 | |
| RBC(>=4*10^12/L) | 0.68(0.27-1.76) | 0.432 | 0.68(0.27-1.76) | 0.432 | |
| Hb(<120*g/L) | 0.62(0.21-1.83) | 0.383 | 0.62(0.21-1.83) | 0.383 | |
| PLT(<100*10^9/L) | 0.79(0.34-1.83) | 0.581 | 0.79(0.34-1.83) | 0.581 | |
| ALT(>=40U/L) | 1.03(0.56-1.88) | 0.924 | 1.03(0.56-1.88) | 0.924 | |
| AST(>=40U/L) | 1.78(0.99-3.18) | 0.053 | 1.78(0.99-3.18) | 0.053 | |
| PT(>=13s) | 1.18(0.66-2.09) | 0.574 | 1.18(0.66-2.09) | 0.574 | |
| TB(>=17.1 umol/L) | 1.88(0.95-3.7) | 0.068 | 1.88(0.95-3.7) | 0.068 | |
| ALB(<40 g/L) | 0.79(0.44-1.43) | 0.445 | 0.79(0.44-1.43) | 0.445 | |
| ALP(>=100U/L) | 1.19(0.67-2.13) | 0.559 | 1.19(0.67-2.13) | 0.559 | |
| AFP(>=400ng/mL) | 2.77(1.38-5.57) | 0.004 | 2.77(1.38-5.57) | 0.004 | |
| CEA(>=5ng/mL) | 1.86(0.74-4.65) | 0.184 | 1.86(0.74-4.65) | 0.184 | |
| CA19-9(>=40U/mL) | 1.67(0.9-3.12) | 0.105 | 1.67(0.9-3.12) | 0.105 | |
| Resection type(major resection) | 0.96(0.54-1.73) | 0.899 | 0.96(0.54-1.73) | 0.899 | |
| Lymph node dissection(yes) | 1.27(0.59-2.72) | 0.536 | 1.27(0.59-2.72) | 0.536 | |
| Intraoperative blood loss(>=200mL) | 0.8(0.45-1.43) | 0.459 | 0.8(0.45-1.43) | 0.459 | |
| Intraoperative blood transfusion(yes) | 0.95(0.47-1.92) | 0.879 | 0.95(0.47-1.92) | 0.879 | |

MiVI,microvascular invasion;HBV, hepatitis B virus; HCV, hepatitis C virus; RBC,red blood cell; Hb, hemoglobin; PLT, platelet count; NLR, neutrophils/lymphocytes ratio; AST, aspartate aminotransferase; PT, prothrombin time; TB,total bilirubin ; ALB,albumin ;GGT,gamma-glutamyl transpeptidase; ALP,alkaline phosphatase ;AFP,alpha-fetoprotein; CEA,carcinoembryonic antigen; CA19-9,carbohydrate antigen 19-9; DCP,decarboxylic prothrombin;AJCC, American Joint Committee on Cancer; CI, confidence interval; OR,odds ratio.

Table S2: Univariable and multivariable cox regression analysis for OS.

| Variables | Univariable cox analysis | | Multivariable cox analysis  based on two-tiered MiVI scheme | | Multivariable cox analysis  based on three-tiered MiVI scheme | |
| --- | --- | --- | --- | --- | --- | --- |
|  | OR (95% CI) | P-value | OR (95% CI) | P-value | OR (95% CI) | P-value |
| Gender(male) | 0.77(0.44-1.35) | 0.369 |  |  |  |  |
| Age(>=65 years) | 0.51(0.26-0.99) | 0.046 | 0.58(0.29-1.15) | 0.12 | 0.56(0.28-1.13) | 0.104 |
| HBV(yes) | 1.5(0.78-2.91) | 0.225 |  |  |  |  |
| HCV(yes) | 0.75(0.1-5.39) | 0.774 |  |  |  |  |
| Cirrhosis(yes) | 1.25(0.79-1.98) | 0.336 |  |  |  |  |
| Tumor number(multiple) | 1.08(0.66-1.75) | 0.766 |  |  |  |  |
| Tumor size (>=5cm) | 1.83(1.17-2.87) | 0.008 | 1.18(0.69-2.01) | 0.547 | 1.15(0.67-1.97) | 0.611 |
| Tumor capsule(incomplete) | 2.38(1.48-3.83) | <0.001 | 2.49(1.53-4.05) | <0.001 | 2.48(1.52-4.03) | <0.001 |
| Microvascular invasion(present) | 1.69(1.07-2.68) | 0.024 | 1.69(1.04-2.76) | 0.035 |  |  |
| Microvascular invasion three-tiered grading |  |  |  |  |  |  |
| M1 vs M0 | 1.37(0.82-2.29) | 0.225 |  |  | 1.54(0.91-2.62) | 0.111 |
| M2 vs M0 | 2.42(1.4-4.19) | 0.002 |  |  | 2.07(1.1-3.9) | 0.024 |
| Macrovascular invasion(present) | 1.85(1.17-2.92) | 0.009 | 1.31(0.8-2.16) | 0.285 | 1.25(0.75-2.08) | 0.401 |
| Lymph node metastasis (present) | 1.43(0.75-2.7) | 0.275 |  |  |  |  |
| Satellite nodules(present) | 1.64(1.07-2.53) | 0.024 | 1.35(0.84-2.15) | 0.212 | 1.24(0.75-2.04) | 0.406 |
| Edmondson-Steiner classification(III/IV) | 0.74(0.47-1.17) | 0.2 |  |  |  |  |
| RBC(>=4*10^12/L) | 0.62(0.25-1.54) | 0.303 |  |  |  |  |
| Hb(<120*g/L) | 0.96(0.39-2.37) | 0.924 |  |  |  |  |
| PLT(<100*10^9/L) | 1.12(0.58-2.16) | 0.745 |  |  |  |  |
| ALT(>=40U/L) | 0.95(0.59-1.51) | 0.822 |  |  |  |  |
| AST(>=40U/L) | 0.76(0.49-1.19) | 0.235 |  |  |  |  |
| PT(>=13s) | 0.97(0.62-1.5) | 0.88 |  |  |  |  |
| TB(>=17.1 umol/L) | 1.18(0.74-1.89) | 0.494 |  |  |  |  |
| ALB(<40 g/L) | 1.18(0.75-1.86) | 0.467 |  |  |  |  |
| ALP(>=100U/L) | 1.65(1.07-2.53) | 0.022 | 1.09(0.67-1.77) | 0.739 | 1.11(0.68-1.82) | 0.664 |
| AFP(>=400ng/mL) | 1.85(1.19-2.89) | 0.006 | 1.02(0.6-1.72) | 0.946 | 1.01(0.6-1.7) | 0.971 |
| CEA(>=5ng/mL) | 1.18(0.62-2.22) | 0.616 |  |  |  |  |
| CA19-9(>=40U/mL) | 2.21(1.43-3.41) | <0.001 | 1.66(1.02-2.71) | 0.043 | 1.63(1-2.67) | 0.051 |
| Resection type(major resection) | 1.69(1.06-2.72) | 0.029 | 1.41(0.83-2.41) | 0.207 | 1.4(0.82-2.38) | 0.217 |
| Lymph node dissection(yes) | 1.19(0.67-2.12) | 0.553 |  |  |  |  |
| Intraoperative blood loss(>=200mL) | 1.23(0.79-1.92) | 0.365 |  |  |  |  |
| Intraoperative blood transfusion(yes) | 1.13(0.65-1.95) | 0.668 |  |  |  |  |

MiVI,microvascular invasion; HBV, hepatitis B virus; HCV, hepatitis C virus; RBC,red blood cell; Hb, hemoglobin; PLT, platelet count; NLR, neutrophils/lymphocytes ratio; AST, aspartate aminotransferase; PT, prothrombin time; TB,total bilirubin ; ALB,albumin ;GGT,gamma-glutamyl transpeptidase; ALP,alkaline phosphatase ;AFP,alpha-fetoprotein; CEA,carcinoembryonic antigen; CA19-9,carbohydrate antigen 19-9; DCP,decarboxylic prothrombin;AJCC, American Joint Committee on Cancer; CI, confidence interval; OR,odds ratio; OS, overall survival.
